# The future of medical scribes documenting in the electronic health record: results of an expert consensus conference

**DOI:** 10.1186/s12911-021-01560-4

**Published:** 2021-06-29

**Authors:** Sky Corby, Keaton Whittaker, Joan S. Ash, Vishnu Mohan, James Becton, Nicholas Solberg, Robby Bergstrom, Benjamin Orwoll, Christopher Hoekstra, Jeffrey A. Gold

**Affiliations:** 1 Division of Pulmonary and Critical Care Medicine, School of Medicine, Oregon Health and Science University, 3181 SW Sam Jackson Park Rd, Portland, OR 97239-3098 USA; 2 Department of Medical Informatics and Clinical Epidemiology, School of Medicine, Oregon Health and Science University, Portland, OR USA; 3 Division of Pediatric Critical Care, School of Medicine, Oregon Health and Science University, Portland, OR USA

**Keywords:** Medical Scribes, EHR, Qualitative Research, Content Analysis Approach, Consensus Conference

## Abstract

**Background:**

With the use of electronic health records (EHRs) increasing and causing unintended negative consequences, the medical scribe profession has burgeoned, but it has yet to be regulated. The purpose of this study was to describe scribe workflow as well as identify the threats and opportunities for the future of the scribe industry.

**Methods:**

The first phase of the study used ethnographic methods consisting of interviews and observations by a multi-disciplinary team of researchers at five United States sites. In April 2019, a two-day conference of experts representing different stakeholder perspectives was held to discuss the results from site visits and to predict the future of medical scribing. An interpretive content analysis approach was used to discover threats and opportunities for the future of medical scribes.

**Results:**

Threats facing the medical scribe industry were related to changes in the documentation model, EHR usability, different payment structures, the need to acquire disparate data during clinical encounters, and workforce-related changes relevant to the scribing model. Simultaneously, opportunities for medical scribing in the future included extension of their role to include workflow analysis, acting as EHR-related subject-matter-experts, and becoming integrated more effectively into the clinical care delivery team. Experts thought that if EHR usability increases, the need for medical scribes might decrease. Additionally, the scribe role could be expanded to allow scribes to document more or take on more informatics-related tasks. The experts also anticipated an increased use of alternative models of scribing, like tele-scribing.

**Conclusion:**

Threats and opportunities for medical scribing were identified. Many experts thought that if the scribe role could be expanded to allow scribes to document more or take on more informatics activities, it would be beneficial. With COVID-19 continuing to change workflows, it is critical that medical scribes receive standardized training as tele-scribing continues to grow in popularity and new roles for scribes as medical team members are identified.

## Background

With the implementation of the HITECH Act, there has been a rapid development and use of electronic health records (EHRs) [1]. This increased expansion in EHR use has been associated with several negative, unintended consequences. One of the most prominent unintended consequences of the EHR is the significant and negative impact on provider workflow due to documentation burden, which is driven by a combination of EHR design and regulatory requirements [2]. Documentation burden is substantial: provider notes in the United States (U.S.) are nearly four times longer than in other countries [3]. To combat these issues with the EHR, organizations have tried a variety of approaches from voice dictation to improved provider EHR training. Another widely adopted solution to EHR inefficiencies and provider burnout has been the incorporation of medical scribes into provider workflow [4].

Scribes have been used for centuries to aid in documentation. Medical scribes were first introduced into medicine in 1975 as nurse-scribes who helped the provider with recordkeeping on patients [5]. With the push towards EHRs, the scribing industry has grown exponentially in health care. The current estimate is that there are nearly 100,000 scribes employed in the U.S. [6]. A medical scribe is typically an unlicensed paraprofessional, often a pre-professional student (typically a pre-medical student), a medical assistant (MA), a registered nurse (RN) or a licensed practical nurse (LPN), whose purpose is to document patient encounters in real time in the EHR and be a part of the clinical team [7]. Prior research has shown that medical scribes can decrease the documentation time for providers and allow providers to spend more quality time with patients [7–11]. Medical scribes lessen the burden of record keeping and order entry aspects of the chart for providers. In addition, scribe notes are also reported to be of higher quality than non-scribed notes [7, 12].

Despite evidence of the positive impact of scribes, several reports suggest potential issues with the rapid expansion of the scribing industry. One concern is the concept of “functional creep” or the increasing responsibilities given to scribes beyond standard documentation, such as order entry and information gathering [13]. Further, while studies suggest scribe notes are more readable, there are concerns about the veracity of data documented in scribe-generated notes [12, 14]. Scribes could also have negative impacts on the provider; for providers to have a scribe, many of them must see more patients and thus increase patient volume to offset the cost of scribes [15]. By seeing more patients, the provider could experience symptoms of burnout despite having the scribe to aid in the documentation. Finally, there is the concern that scribes are a merely a “Band-Aid solution” and fixing EHR usability is critical; an increased use and reliance on medical scribes could result in a slower development of a more capable EHR system [13]. Many of these concerns are not evidence-based because there is a large gap in our knowledge about scribe activities in the U.S.. To identify present practices, the research team conducted site visits around the U.S. to determine the scribes’ workflow and their impact on health systems [16]. These site visits were to develop lists of needed knowledge, skills, and attitudes of scribes (KSAs) and a standardized training program for scribes so they could become more efficient at their jobs.

Given the rapid growth of scribing and continual evolution of their role, in order to assess training needs, the next step aimed to verify results with experts and to identify threats and opportunities facing the future of the scribe industry.

## Methods

The research team held an in-person conference for experts to review the results from the site visits and discuss the future of scribing.

### Pre-conference activities

The goal of the parent study, funded by the Agency for Healthcare Research and Quality (AHRQ), was to create lists of KSAs and develop a training program for medical scribes with workflow efficiency and patient safety in mind. From October 2017 through January 2019, the research team conducted five site visits throughout the U.S. to investigate medical scribes. A qualitative approach, the Rapid Assessment Process (RAP) model, a widely accepted qualitative research method, was utilized. RAP is a quick and efficient way to gather qualitative data using a team-based approach [17, 18]. Triangulation was achieved by a multidisciplinary team through intensive, iterative review of audio-recorded semi-structured interviews, and site visit observations documented in field notes. With NVivo 11, researchers coded the transcripts and observation field notes using an inductive grounded theory approach. Full details of the methodology and analysis have been previously published [16, 19, 20].

Using a modified Delphi approach, a two-day expert consensus conference was held to discuss the findings from the site visits and review the topology of scribe utilization in healthcare, with the aim of developing knowledge, skills, and attitudes medical scribes should possess [21–23]. Prior expert conferences, similar to this one, had yielded informative results [24, 25]. The research team held a two-day conference in April 2019, which included twenty subject-matter experts from across the U.S. with varying professional backgrounds. More specifically, those invited were a mix of representatives from important stakeholder groups: medical scribe vendors, informaticians, risk managers, former medical scribes, CMIOs, representatives from accreditation bodies, providers, and social scientists [26, 27]. Prior to the conference, the subject-matter experts reviewed the results of the research team’s site visits prior to the conference. During the conference, there was a robust discussion about the future of scribing with attention to threats and opportunities for the industry. To our knowledge, no prior research has investigated scribe utilization and what the landscape of scribing might look like in the future.

### Conference activities

On the first full day, the principal investigators led a discussion on result findings, simulation-based learning, and the creation of the KSA lists. Next, a trained facilitator had everyone break into small groups (predetermined to allow for different perspectives in each group) and answer one of the “Five Big Questions” surrounding medical scribes. The five questions were: (1) What are the patient safety risks of using scribes and how great are they? (2) How much standardization of documentation is possible, given the variation in provider preferences? (3) What should the scope of practice related to the EHRs include, in addition to documentation? (4) How good is the quality of documentation and coding done by scribes? and (5) What are the downsides to an organization hiring scribes? The group then later reconvened to discuss responses for each of the group’s question. Next, the groups received lists of knowledge, skills, and attitudes of scribes related to the EHR (results of the site visits) and broke into small groups that later met as a larger group to discuss the lists as a whole. The last activity was a free response activity where everyone told one or more stories about medical scribes. During these activities, the participants learned about each other’s perspectives and the group developed a shared knowledge about scribing.

On the final day of the conference, designed to take advantage of group synergy and creativity, every small group reviewed designated lists related to general knowledge, skills, and attitudes scribes should possess that were not EHR related. After the small group session, everyone met to discuss their lists as a larger group. The final activity of the conference was to discuss the future of scribing, specifically threats and opportunities for scribing in the future. For this part of the conference, there were two flipcharts with threats written on one and opportunities written on the other. Participants took turns discussing what they thought the threats to scribing in the future entailed and what the opportunities were. One team member was in charge of writing down the opportunities of scribing and one of the experts was in charge of writing down the suggestions for threats to scribing.

The researchers will summarize the threats and opportunities of medical scribes in the future related to EHR use discussed by the experts and based on the site visit results.

### Post-conference activities

Small group and large group discussions were recorded and audio files were transcribed. Multiple individuals at the research conference recorded the threats and opportunities of scribing discussion. Because of this, the research team resulted in multiple transcripts of the same recording, each with some missing elements. One member of the team triangulated all the transcripts and audio files and created one document that was as complete as possible.

Analysis: Using NVivo 12, team members captured the bullet points written on the flipcharts for the threats and opportunities lists as a basic codebook. There were two main codes for future of scribing: (1) opportunities and (2) threats. For opportunities, there were multiple subcodes: (1) artificial intelligence, (2) different payment models, (3) informatician role, (4) nursing assistance, (5) opportunity cost, (6) patient entered data, (7) reimbursable scribing, (8) remote scribing, (9) structured data, (10) teamwork and present boundaries, and (11) workflow. For threats, the subcodes were: (1) artificial intelligence, (2) broader documentation model, (3) decreased workforce, (4) delay of patient care, (5) different clinical situations, (6) different payment models, (7) funding model, (8) internal scribe company, (9) livable career, (10) patient entered data, (11) provider retirement, (12) regulation, and (13) remote scribing. The researchers reviewed the transcript and any time one of these codes or subcodes was used, they would code them in NVivo. The researchers then conducted a thematic content analysis. Content analysis is defined as a type of narrative analysis that uses a “systematic coding and categorizing approach used for exploring large amounts of textual information unobtrusively to determine trends and patterns of words used, their frequency, their relationships, and the structures and discourses of communication” [28 p. 400].^.^ The team members systematically reviewed the transcript and looked for meaningful quotes related to the bullet points written on the flipcharts at the conference. The researchers identified various themes across the codes, which are depicted in the results.

## Results

**Fig. 1:**
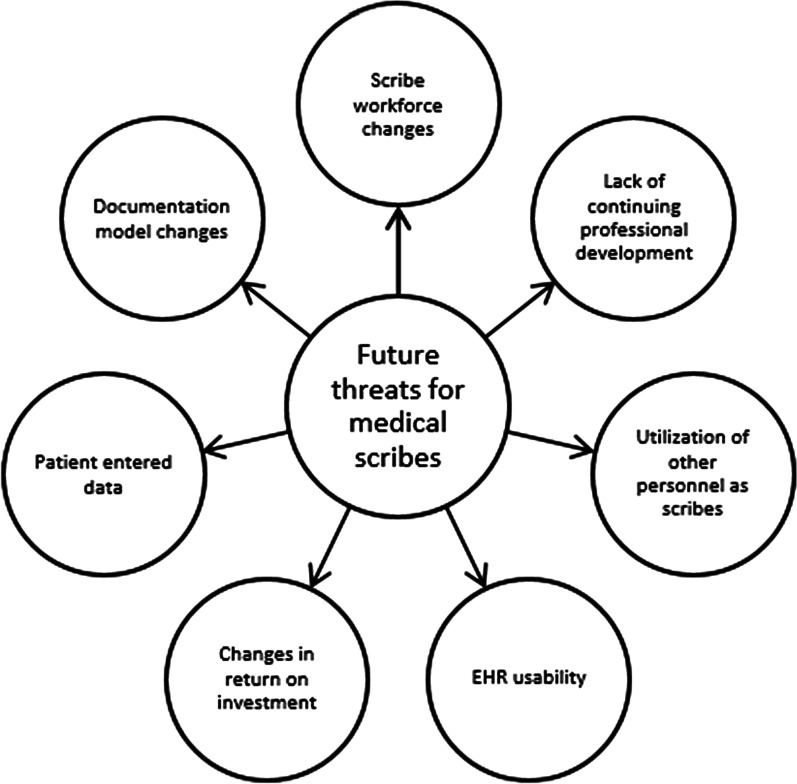
Threats to future of medical scribes

All participants actively contributed to the discussion. There was consensus that the threats to the medical scribe industry include the themes of: changes in the documentation model (including artificial intelligence), changes in the scribe workforce, lack of opportunities for continuing professional development, utilization of other personnel as scribes (MAs/LPNs/RNs), EHR usability, possible changes in the return on investment case for scribes, and increased use of patient entered data (Fig. 1).

### Threats for the future of scribing

#### Change in documentation model

One threat to the future of scribing the experts and team members described was the need for a better documentation model. Changes in documentation that allow visits to be recorded with audio and/or video, and therefore enable visits to be archived, would allow providers to review patient visits and revamp notes without the need for medical scribes. However, technology and regulatory needs are not able to adopt to this model currently.

One expert noted that, “*there will be some interesting things like voice-to-text and that artificial intelligence could help us go through things and for example come up with a skeleton of the note which people could then populate and that would probably be better than what we are doing today.*” With documentation changes and the evolution of artificial intelligence, there may not be a need for medical scribes to do those aspects of the chart anymore.

Another expert noted that in the U.S., provider notes are four times longer than other countries; providers in those other countries are more satisfied with EHR use than providers in the U.S.. He went on further to say that, “*the regulations around which you have to include in your documentation to get paid, could be a factor and should probably be revised.*” If regulations change so providers’ notes are less lengthy, it could allow providers to be more satisfied with the EHR and potentially feel less burned out and less likely to need a scribe, according to the group. In summary, because the role of a scribe is to facilitate documentation, if regulations on documentation change, then the need for a medical scribe would likely shift as well.

#### Changes in the scribe workforce include the limited availability of pre-professional scribes

Another potential threat to the future of scribing would be a decrease in the availability of talented pre-professionals. Typically, this pre-professional model utilizes undergraduate college students who want to go into the healthcare field. One expert questioned that if the rise of medical scribing continues, “*are you going to continue to get high-quality, pre-professional students to fulfill this role, or are you starting to get the lower end of the bell-shaped curve?*” If demand for scribes outstrips the supply of pre-professional candidates, it is possible that this current scribing job model will have to be adjusted.

Along those same lines, an informatician expert wondered that, “*if you are in a small town, we don’t have a medical school, you aren’t going to have these people. So, are we always going to have these two tracks? Are we going to have enough of the medical school people? If not, are we able to build a middle-class job that people will be able to sustain their life on as a scribe in the future?*” This emphasizes that the need for scribes beyond a pre-professional model might be warranted.

#### Role may not offer opportunities for continuing professional development

Another aspect to this is the idea that scribing could become a less valuable stepping-stone for medical scribes to further their future professional development. One of the team members was curious that, “*if it is not a value added are they going to be willing to suffer the indignities of doing this for 18 months because of the way they are treated and paid?*”.

Another expert noted that, “*if we are going to have one hundred thousand kids coming out of college wanting to be scribes, eventually you’re A, going to not get people who are as capable of doing it, and B, if it doesn’t help them get into medical school… then no one is going to be a scribe as a way to get in [to medical school].*”

Because of this, other models of scribing might have to be used, including professional scribes with clinical training.

#### Utilizing other personnel as scribes (MA/LPN/RN)

As mentioned above, using other models of scribing, like MAs/LPNs/RNs as scribes could be utilized, which is a threat to the current popular model of scribing, the pre-professional model. Some organizations use a two MAs per doctor model where “*they are leapfrogging and so they do chart prep organization, order pending, health maintenance, what you need to get done at the visit.*” MAs who scribe could have a pay incentive if they agreed to include scribing as a part of their duties.

Another advantage of using MAs/LPNs/RNs as scribes is that these professions are already a part of the organization’s culture. Some pre-professional scribes are in-house and hired as part of the organization, but not all. Some use third-party scribe companies to hire scribes. One person mentioned that when she was a pre-professional scribe for a third-party scribe company, the organization she worked for treated her like an outsider. She said that, “*[scribes] don’t have an allegiance or they don’t have the right training for that organization. As the organization [rolls] out new initiatives, *etc*. when you are an employee of that organization you are a part of that. When you are outside of it you are there to serve that doctor and you can kind of view it as, “oh, that’s them, I’m not gonna do it,” but when you are hired and you have a supervisor in a clinic it’s a different thing.*” This can create a silo effect, which can occur when a team does not communicate effectively or work together to solve problems, resulting in workflow inefficiencies. If a scribe works for a third-party scribe company, that scribe may not collaborate and communicate effectively because they are not fully socio-politically incorporated in the organization. However, with the MA/RN/LPN model, the people scribing would already be employees of the organization and have insight into workflows and procedures and would already be a part of the team and minimize this silo effect. One expert said that, “*it’s really important to think about a broader model and that broader model from everything that I have seen is going to be best when you consolidate roles into a small number of people. And I think two MA or two RN, two physician model in my view is a very effective opportunity.*”

The disadvantage to this model is that clinical scribes, like MAs/RNs/LPNs, may not be suited for the scribe role, plus they would cost more per hour than a pre-professional scribe, especially if they are getting a pay incentive to do scribing activities. However, some organizations are using “*what they already have in house [i.e. MAs, RNs, or LPNs]*” as scribes because of the convenience.

#### EHR usability

Some experts noted that the job of the scribe will change if EHRs change. Right now scribes are “*band aiding [the] EHR.*” Another expert noted that*,* “*If the EHR becomes more user-friendly… then we have to determine what the need for a scribe is.*” The need for the role of scribing may become less pertinent if the EHR becomes more user friendly.

#### Changes in the return on investment case for scribes

Different payment models are a threat to the future of medical scribes as well. In the traditional pre-professional model of scribing, most organizations use a return on investment (ROI) based on the number of patients seen. This means that providers who want to hire scribes must be willing to see a certain number of additional patients per day to offset the cost of hiring a scribe. In the traditional pre-professional scribing model, the model is “*built on unlivable wages*” for scribes to make them cost effective for organizations.

Medical scribes are not paid enough to be able to live comfortably, which could contribute to the high turnover rate of medical scribes in the pre-professional model. One person said that “*yes, we could pay scribes more. It would retain them [to last] 10 years. But if you are running your own scribe company or [hiring them] personally, then [the provider would] have to see more patients.*”

Seeing more patients could cause providers to be even more stressed and burned out. Another option for a different payment model would be “*if we change it to make scribing reimbursable–- because that is the whole reason we have to keep scribe wages low. They just add to the overhead right now. They’re not bringing in any additional money other than secondary. All of that is still billed through the provider. So, a potential opportunity for me is if we have a different payment model that actually starts reimbursing the scribe for their role that would potentially allow for more livable wages and a more professional model where someone stays and that’s their career.*”

#### Patient entered data

The last potential threat to the future of scribing could be patient entered data. Allowing patients to enter some of their own data could allow for organizations to “*deliver healthcare more effectively with more accurate story from the patient.*” If patients are able to enter data and document before the office visit, the need for a medical scribe could be limited. Some organizations are already experimenting with patient entered data.

Overall, we found that there are several threats affecting the scribing industry (Table 1).

**Table 1:** Threats to medical scribe industry

Threats to medical scribe industry		
Types of threats	Summarization	Example
Change in documentation model	Various changes in documentation model, based on regulation and technology advancements, could lead to not needing medical scribes to aid in documentation	Using artificial intelligence as a way to help with documentation
Change in the scribe workforce	The pre-professional model is the most popular model of medical scribes. However, with the increased use in medical scribes, different models may have to be utilized	Using non-medical school bound individuals as scribes
Lack of opportunities for continuing professional development	Being a pre-professional medical scribe may not be beneficial when applying to medical schools	A medical scribe being paid poorly, working very hard, but still not getting into medical school programs
Utilizing other personnel as scribes (MA/LPN/RN)	Using people in house to be documenters for the providers	Using LPNs or MAs or RNs as scribes
EHR usability	Medical scribes are just a band-aid solution to the EHR. With more EHR ingenuity, the need for medical scribes could decrease	Optimal design of documentation inside the EHR
Changes in the return on investments case for scribes	Changing the pay model of pre-professional scribes from a human ROI model to a reimburseable model	Making scribing reimbursable for providers
Patient entered data	Allowing patients to enter data into their chart	OpenNotes

#### Opportunities for the future of scribing

**Fig. 2:**
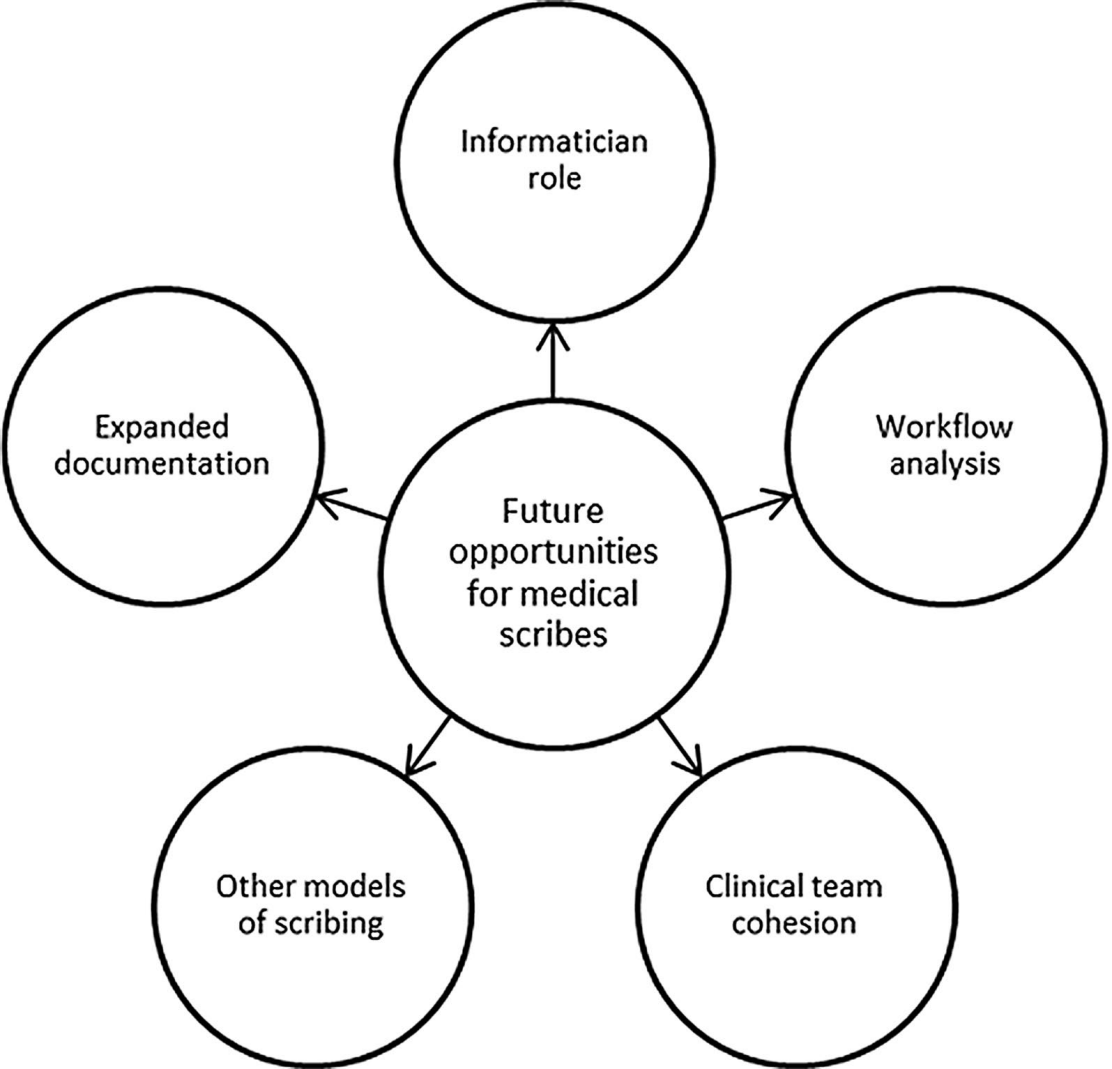
Opportunities for the future of medical scribes

During the conference, team members and experts discussed what the future of scribing could ultimately look like and themes were formed during the analysis (Fig. 2).

The themes for opportunities for the future of scribing were: allow scribes to document more, train them to play an information technology analyst role, extend the role to include workflow analysis, extend their duties to improve clinical team cohesion, and the development expanded models of scribing.

#### Allow scribes to document more

Allowing scribes to collect a broader range of notes and documentation styles was mentioned as an opportunity. One expert stated that, “*I think a huge opportunity for scribes is to transition to new documentation templates or ways to collect data more discreetly. Whether it be an actual point-and-click data collection form or making the note smarter.*”

Scribes could also guide providers towards better documentation strategies. One expert stated that it would be useful to identify “*those scribes who are just really [tech] savvy… and connecting them with the clinical and informatics or IT department.*” Thus, medical scribes could help providers who have “*been using this kind of bad note for 20 years*” and scribes could update the note template and show the provider other strategies to help them work more efficiently.

#### Informatics role

Another opportunity for scribes could be to allow them to practice more information management. There could be a continuum where scribes “*could move up, they could do order entry [and medication reconciliation]… if scribes could do that it would be a big win for scribes.*” Additionally, if regulatory changes could be made so medical scribes could have further responsibilities, “*they can be a value in the future.*” Allowing for a continuum where scribes, over time, could increase their job responsibilities and could further decrease the provider’s workload.

#### Extend role to include workflow analysis

An opportunity for medical scribes would be to extend their role to include workflow analysis aspects. One expert said that “*because of my background and experience [as a scribe], it is very easy for me to get it and write a good note—I’m like, “Your idea is great, doctor. We’re not going to do that. I have the street cred to tell them why and how to get them to navigate faster and write a better note.*” Organizations could hire scribes to be workflow analysts, but could offer them less pay than someone would for a physician or nurse informaticians. This could be a new role for medical scribes.

#### Improve clinical team cohesion

Another opportunity could involve the inclusion of having the scribe be more part of the team. One expert put forward, “*There is all this team work that needs to be done because physicians are doing non-doctor work 80% of the time. So, how do we build [it]? If we are going to use people, not young people who we can pay cheap and who don’t burnout because they are only there for 3 months, who both find a job that is interesting and fulfilling, they become part of a team, they have self-worth. Can we build that as a scribe position or not?*”

The experts and team members discussed some of the regulatory issues regarding medical scribes (i.e. scribes not being able to help patients off the bed due to liability if the patient or scribe gets injured). Someone stated that, “*I’ve really had trouble when we talked about the scribe [who] couldn’t help the patient onto the table and I understand the problems with insurance and things, but if that was my clinic and the nurse saw that the scribe wouldn’t touch a patient it would be like, “we all kind of work together here” and I understand there are different parts to it but I think what we have come to is to have a fulfilling job in healthcare you become part of a team and you feel valued.*”

Allowing scribes to have an extended role would enable scribes to increasingly be a part of the whole health care team and allow them to help with tasks that existing standards do not allow at the moment.

#### Other models of scribing

Another opportunity for the medical scribing industry as a whole would be to expand the role and utilize different models of scribing. Some organizations are moving away from the traditional third-party scribe company vendor model and moving towards two MAs or two RNs. Moving to this model can save the providers more money than they would have if they chose a pre-professional scribing model. One person discussed a model they “*have had in [their] practice with two and a half nurses per physician for years and years. It’s the model that [they] mentioned to [us] that [XXX] Health has that one hundred of their one hundred and fifty physicians are now in that model. They have the top scores for the [XXX] ACO out of the forty [XXX] ACOs, they had the best. It’s saved them $27 per member per month.*”

Another model of medical scribes that has increased in popularity is tele-scribing. One expert mentioned that tele-scribing centers will become the next call centers, where the scribes might not be located inside the U.S., but could be “*in another country where it’s even a lower value of work force and quality control.*” Tele-scribing would allow providers who live in rural areas some with much needed assistance. One scribe vendor said that, “*There is such a shortage of specialists and so making those specialists very effective by pairing them with somebody to support them in the documentation is a big focus right now [for all scribe vendors].*”

Tele-scribing provides greater scribe accessibility. It is the providers in the smaller and more rural hospitals who are getting burned out, but their “*patients are waiting to see their providers.*” One expert noted that the “*slow adoption of this resource is a big threat to patient care because so many of these areas have access issues…. [there should be] cost [e]ffective ways to do this so that the adoption rate goes up a lot and if we want to take care of our providers we’ve got to help them or they are going to continue to burn out.*”

Tele-scribing has potential, but attendees at the conference mentioned that instead of overseas scribes, they envisioned scribes located in the U.S. but working remotely rather than on site. One expert said, “*it actually eventually leverages in matching where I have people that have the capacity with the places where it’s needed. So, it might be West Coast versus Mid-West or it might be people in Alaska or it might be the regional hospital where it makes no financial sense for someone to drive up and see the patient.*”

Summarization of opportunities of scribing can be seen in Table 2.

**Table 2:** Opportunities for medical scribes

Opportunities for the medical scribe industry		
Type of opportunity	Summarization	Example
Allow scribes to document more	Having scribes be able to do more documentation, including updating note templates and collecting data more discreetly	Teaching providers how to improve documentation
Informatician role	Have scribes have more of an information management role	Scribes could do order entry and medication reconciliation
Extend role to include workflow analysis	Scribes work very closely with the providers and could provide insight for the organization on how providers could write more efficient notes	Having scribes team up with workflow specialists to help providers figure out a way to write a better note
Improving clinical team cohesion	Some mentioned it would be great if the scribe could be more a part of the health care team as a whole	Having the scribes being able to assist patients on/off the bed, handing the patient the AVS, showing the patient out of room
Other models of scribing	Using other models of scribing would move things away from the pre-professional model but could create longer lasting scribing roles	Using MAs/RNs/LPNs or telescribing model

## Discussion

This study is the first to the research team’s knowledge to describe the threats and opportunities related to the future of medical scribes. This knowledge will help our team develop training for scribes that prepares them for future roles. It will also contribute to the evidence-base upon which organizations and the scribe industry can make decisions about scribe training.

The experts described a multitude of threats to the future of scribing which can be placed in two categories: External pressures on the system and internal pressures on the system. For external pressures, experts noted that as EHR usability improves, the need for all medical scribes, regardless of the model, could decrease. Changes in wages and reimbursement models, as well as allowing patients to enter data into the chart, could change the future of medical scribing. The medical record documentation burden has already decreased for many services following the Centers for Medicare and Medicaid Services (CMS) coding changes in January 2021 [29]. With reduced documentation requirements for this aspect of the record, the need for medical scribes may become unnecessary for some providers. We learned that artificial intelligence and video/audio recording patient visits could lead to changes in the documentation model if regulatory and technology advances continue. Research has shown that patients are entering their own data into the EHR/Patient Portal as well. The authors of one study noted that patients were able to use an app on their phone to track asthma health and connect it to their patient portal app, which allowed the providers to review the patient entered data and submit orders for medication [30]. In another study, the authors conducted a systematic review on patient generated health data and reported that having patients entering their own data could increase patient’s awareness and the communication between the provider and patient [31]. A different study noted that because of COVID-19 and the rapid shift to telemedicine, patients were entering their biometric information into their patient portals; the authors provided an example that pregnant women with hypertension were syncing their home monitoring devices to their providers through the EHR [32].

We also saw internal pressures to the system. The most notable of these could be the increased use of alternative models of scribing. This could entail the increased employment of non-medical school bound individuals or MAs/LPNs/RNs, which would decrease the need for the popular pre-professional scribing model. As the use of other models of scribing grows, medical scribing might no longer be a stepping-stone to professional schools, which would fundamentally change the size and skills of the workforce. This would also deprive recent college graduates of the opportunity to gain documentation skills and knowledge of the culture of healthcare before applying to medical school. One study discussed how being a scribe allowed scribes to start thinking like a provider by having them understand the reasoning behind decision-making and what to look for on physical exams [33]. Being a scribe also teaches professional development like organizational skills, how to function as a team, and interpersonal communication skills [33]. These authors state that no other shadowing opportunity allows pre-medical students to be as immersed in medicine as scribing. In a qualitative study, it was noted that medical students who were scribes and exposed to medical terminology had an advantage upon entering medical school compared to their counterparts [34].

There were multiple opportunities for scribes in the future as well. We saw these through the lens of external and internal pressures on the systems. From external pressures from the system, we saw that the scribe role could be expanded to allow scribes to document more as well as take on more of an informatician role or workflow analysis role. If CMS and other regulatory/certifying bodies, such as Joint Commission and other medico-legal pressures and institutional policies, could expand the role of medical scribes, which would allow scribes to become more of a significant part of the health care team. In a quantitative study at a large academic hospital, the researchers reported that scribes have taken on some new roles due to COVID-19. Some scribes are doing pre-charting only, remote scribing for virtual visits, and remote scribing for traditional in person visits [35]. Due to redeployment, scribes are aiding in non-visit-related tasks like “working in the virtual help desk… check point staffing, and chart abstraction” [35 p 809]. This shows that the scribes’ role could expand to include activities not typically involved in scribing duties. Through examining the internal pressure to the system, we saw the benefit of utilizing other models of scribing, specifically tele-scribing. During the conference, it was noted that tele-scribing allowed providers who are doing in person or virtual patient encounters in remote or rural locations to still have access to scribes. With the pandemic, the push for tele-scribing has increased dramatically in not only rural areas, but also at larger academic hospitals [35]. There was an increase in scribes who were doing tele-scribing, regardless of whether the provider and/or patient were remote as well [35]. Another study showed that because of the COVID-19 pandemic, an otolaryngology clinic went to tele-medicine and therefore their scribes went to tele-scribing [36]. The authors outlined a protocol for scribes so they know how to participate in telemedicine appointments and what their role is during the telemedicine visit.

There are several limitations to this study. Its qualitative nature means only a small number of experts were included. However, these experts were purposively selected for the conference to add to geographic and professional diversity. The researchers invited representatives from diverse stakeholder groups so the researchers could gather differing opinions. Another limitation to this study was regarding the time allowed for the discussion of the future of medical scribes, which was approximately one hour and could be considered an inadequate amount of time. Perhaps a lengthier discussion could have led to more in-depth results and allowed for experts to achieve unanimous consensus before the end of the conference. The lead investigators did try to combat this by scheduling it at the end of the conference after synergy had developed, which made it especially productive. A final limitation was that the conference took place pre-COVID-19. With the rapid change to telemedicine due to the pandemic, tele-scribing could be discussed in more depth at a later date.

The landscape of scribing continues to change, especially with the unpredictable nature of the COVID-19 pandemic. Overall, we recommend increased oversight and the development of guidelines as well as an increase in the standardization of documentation. It is critical that medical scribes receive adequate and standardized training to learn how to do their job effectively, which is the ultimate purpose of the team’s present project. It also would be beneficial to do an analysis of tele-scribing in every potential permutation since it is growing in popularity and little research has been conducted on this model of scribing. Because of tele-medicine’s rapid and potentially uncontrolled growth due to COVID-19, it could lead to a number of significant unintended consequences.

## Conclusions

We have identified future trends in medical scribing, including threats and opportunities, which are important to consider when developing scribe training programs. While expert opinions differed, a common sentiment was that the scribe role could be expanded to include elements to better integrate the scribe into the care delivery team. Allowing scribes to expand their roles could lessen the burden on providers and ultimately add to a more cohesive team environment.

## Data Availability

Data sharing is not applicable to this article as no datasets were generated or analyzed during the current study.
